# Comparative efficacy and safety of traditional Chinese medicine for lipodermatosclerosis: A protocol for systematic review and network meta-analysis

**DOI:** 10.1097/MD.0000000000023386

**Published:** 2020-11-20

**Authors:** Mengmeng Du, Yudong Zhang, Xiaohua Shi, Ming Liu

**Affiliations:** a First Clinical Medical College, Shandong University of Traditional Chinese Medicine; b Department of Peripheral Blood Vessel, The Affiliated Hospital of Shandong University of Traditional Chinese Medicine, Jinan, Shandong; c Department of Trauma Orthopaedics, Zhengzhou Orthopaedic Hospital, Zhengzhou, Henan, China.

**Keywords:** lipodermatosclerosis, network meta-analysis, protocol, traditional Chinese medicine

## Abstract

**Background:**

Lipodermatosclerosis (LDS) is a severe skin change accompanied by the development of chronic venous disease of the lower extremities. Its main clinical manifestations are erythema, induration, hyperpigmentation, and rough and thickened skin. It may also eventually lead to refractory ulcers, skin necrosis and even cancer. Conventional treatment methods mainly include the intake of oral anabolic hormones or androgen and pressure therapy. However, patients often refuse due to their drug resistance and intolerance. As a clinical irreplaceable treatment method for LDS, traditional Chinese medicine (TCM) has not been compared of the safety and effectiveness so far. Therefore, we cannot wait to use a method to compare the efficacy of TCM for LDS systematically, such as network meta-analysis (NMA).

**Methods:**

We will retrieve the relevant Chinese and English databases comprehensively. All the randomized controlled trials of TCM for LDS from January 2015 to September 2020 will be included. Under the guidance of inclusion criteria, 2 researchers will screen the literature, then assess the risk of bias and extract data. We will use Bayesian NMA to evaluate all available evidence in STATA 14.0 and WinBUGS software.

**Results:**

This study will use Bayesian NMA to evaluate the efficacy and safety of TCM for LDS.

**Conclusion:**

This study provide a reliable theoretical basis for the clinical application of TCM in the treatment of LDS, and contribute to the formulation of treatment guidelines for LDS.

## 1 Introduction

Lipodermatosclerosis (LDS), also known as Localized Scleroderma or Dermatosclerosis, is first described in 2009 as the C4b stage of varicose veins of the lower extremities by Shiman et al.^[1]^ Today, it is generally believe that LDS is a part of the pathological progress in the progression of chronic venous disease (CVD).^[2]^ It is a non-bacterial inflammatory reaction of the skin and fat caused by the activation of leukocytes and inflammatory mediators under a background of venous hypertension. Its main clinical manifestations are erythema, induration, hyperpigmentation, and rough and thickened skin. It may also eventually lead to refractory ulcers, skin necrosis and cancer,^[3]^ which negatively affects the quality of life, increases psychological burden, and even enormously threaten patients’ lives.^[4,5]^

CVD is more common in industrialized countries and generally occurs in the middle-aged and older women. A study shows that the incidence of CVD ranges from 10% to as high as 30%, and approximately 10% of CVD patients will develop LDS.^[6]^ Recent research showed that the incidence of LDS connected with ulceration is estimated to be approximately 13%, increasing annually.^[7]^ Another study based on skin disease types in the obese patients found that the incidence rate of LDS in obese population was 0.5%.^[8]^

**Figure 1:**
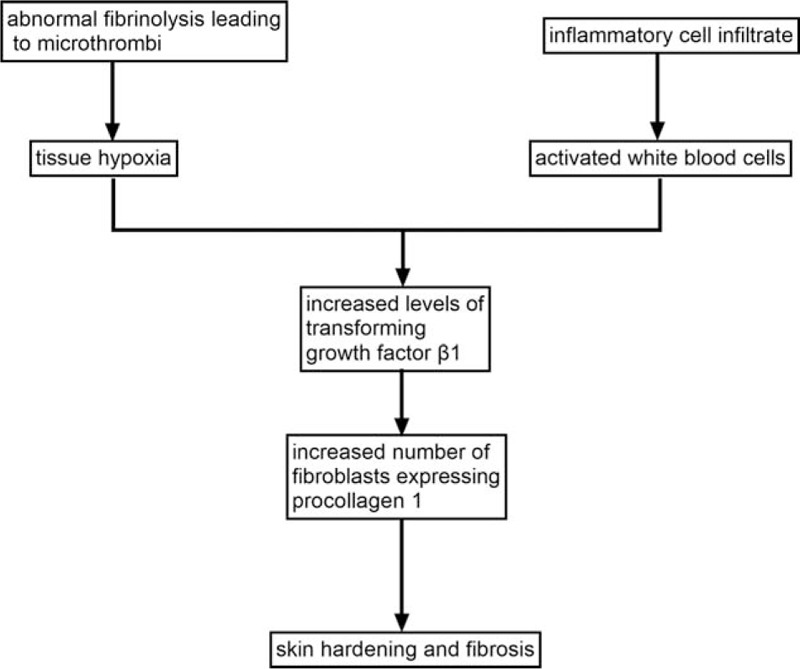
Diagram of the proposed pathophysiology changes in LDS.

Several possible pathophysiological mechanisms of LDS have been proposed in recent years. Abnormal fibrinolysis of LDS eventually leads to localized microthrombus formation, tissue anoxia, and oxygen starvation, which have been discovered to be stimulating factors of TGF-β1. Activation of WBC also increases the level of TGF-β1. TGF-β1 conversely influences collagen production, resulting in fibrosis (Fig. 1). It can be seen that TGF-β1 plays an essential role in the development of LDS.^[7]^

According to reports, the efficiency of various treatment options is controversial. Although compression therapy is the initial recommendation for the treatment of LDS, many patients with stress therapy develop ulcers as the condition progresses.^[9]^ A study^[9]^ demonstrates the beneficial effect of compression stockings on LDS secondary to chronic venous disease, but no benefit on symptoms. Moreover, most patients refused stress treatment because of intolerance. Previous studies of LDS focused on the anabolic steroids, stanozolol and pentoxifylline.^[10–12]^ However, it may be challenging to reduce the side effects associated with this class of medicine, such as androgenic changes, acne, oily skin, weight gain, fluid retention and hypercholesterolemia.^[7]^ Besides, LDS has been reported to be notoriously resistant to the usual local and systemic treatments, the typically used anabolic steroids, stanozolol and pentoxifylline, may only be effective for some patients.^[13]^ Various studies have asserted that the application of antibiotics, anti-inflammatory drugs, anti-metabolic drugs and intravascular interventional therapy has obvious limitations.^[3]^

As a powerful weapon of traditional Chinese medicine (TCM), Chinese herb has its unique nature, taste and meridian tropism. TCM has small side effects and reliable efficacy, plays an vital role in Preventing and treating disease. The pathogenesis of LDS is the same as Localized Scleroderma, mainly including meridians bizu, deficiency of qi and blood, viscera deficiency, phlegm YuZu collaterals, related with the lung, spleen and kidney. TCM treatment based on syndrome differentiation and herbal fumigation can help to reduce the hardening.^[14]^ Basic research has shown that hot compress of herbs could improve skin sclerosis, with apparent curative effect and relative safety.^[15]^

To the best of our knowledge, as a clinical irreplaceable treatment method for LDS, TCM has not been compared of the safety and effectiveness so far. As an extension of traditional meta-analysis, network meta-analysis (NMA) can make full use of clinical data, and compare efficacy of more than 3 treatment methods. Therefore, we will use it to compare the safety and effectiveness of TCM for LDS.

## 2 Materials and methods

### 2.1 Study registration

We will use Bayesian NMA. The implementation of this study follows the PRISMA-P guidelines. We have been registered on the International Platform of Registered Systematic Review and Meta-analysis Protocols (INPLASY) and the registration number is INPLASY2020100090 (URL: https://inplasy.com/inplasy-2020-10-0090/).

### 2.2 Inclusion criteria

#### 2.2.1 Type of study

We will collect all relevant randomized controlled trials (RCTs) of TCM for LDS published in Chinese or English.

#### 2.2.2 Participants

The diagnosis of LDS will follow the guidelines for Lipodermatosclerosis, regardless of age, severity, duration, race or gender.

#### 2.2.3 Interventions

The control group have been treated with western medicine, while the treatment group must have been treated with TCM on the basis of conventional treatment. The course of treatment, dosage and operation were not taken into account.

#### 2.2.4 Outcomes

The outcomes of our interest are chronic venous insufficiency questionnaire (CIVIQ),^[16]^ venous clinical severity score (VCSS)^[17]^ and the level of transforming growth factor-β1 (TGF-β1) in serum, and the details are as follows:

1. According to the method of CIVIQ, the scale of 1 to 5 was adopted. The assessment included social activities, mental and psychological, physical fitness and pain.
2. According to the VCSS, each score was divided into 4 grades: 0, 1, 2 and 3. The main indicators included pain, edema, varicose vein, pigmentation, inflammatory reaction, Scleroderma and ulcer.
3. The level of transforming growth factor-β1 (TGF-β1) in serum.

The literature included must cover one or more indicators above.

### 2.3 Database and search strategy

With the help of an experienced librarian, we have customized search strategies for each database. We will search Web of Science, PubMed, China BioMedical Literature (CBM), EMBASE, Cochrane Library, China National Knowledge Infrastructure (CNKI), and Wanfang database from January 2015 to September 2020, without restrictions for language, or publication status. The search strategy mainly includes Medical Subject Headings (MeSH) and free-text terms, such as “Chinese herbal drugs, Alternative Therapies, Lipodermatosclerosis, Localized Scleroderma, Dermatosclerosis, Randomized controlled trial,” etc. The detailed search strategy of PubMed is shown in Table 1. In addition, we will search the ongoing trial registered on the International Clinical Trial Registration Platform.

**Table 1:** Detailed search strategy for PubMed.

No.	Search item
1#	“scleroderma, systemic”[MeSH Terms]
2#	“Lipodermatosclerosis”[Title/Abstract] OR ((“localized scleroderma”[Title/Abstract] OR “systemic scleroderma”[Title/Abstrac] OR “Scleroderma”[Title/Abstrac] OR “scleroderma circumscribed”[Title/Abstract] OR “circumscribed scleroderma”[Title/Abstract] OR “Dermatosclerosis”[Title/Abstract] OR “Morphea”[Title/Abstract] OR “Morpheas”[Title/Abstract] OR “scleroderma linear”[Title/Abstract] OR “linear scleroderma”[Title/Abstract] OR “scleroderma localized”[Title/Abstract]
3#	1# OR 2#
4#	“Complementary Therapies”[MeSH Terms]
5#	“therapies complementary”[Title/Abstract] OR “therapy complementary”[Title/Abstract] OR “complementary medicine”[Title/Abstract] OR “medicine complementary”[Title/Abstract] OR “alternative medicine”[Title/Abstract] OR “medicine alternative”[Title/Abstract] OR “alternative therapies”[Title/Abstract] OR “therapies alternative”[Title/Abstract] OR “therapy alternative”[Title/Abstract] OR “herbal therapy”[Title/Abstract] OR “herb therapy”[Title/Abstract] OR “chinese patent medicine”[Title/Abstract] OR “chinese proprietary medicine”[Title/Abstract] OR “chinese herbal drugs”[Title/Abstract] OR “Herbal”[Title/Abstract]
6#	4# OR 5#
7#	“randomized controlled trial”[Title/Abstract] OR “controlled clinical trial”[Title/Abstract] OR “Randomized”[Title/Abstract] OR “random allocation”[Title/Abstract] OR “Randomly”[Title/Abstract]
8#	3# AND 6# AND 7#

### 2.4 Study selection and data extraction

Two researchers will conducted literature screening and data extraction independently. Duplicate literatures will be searched by Noteexpress and checked manually. According to the established inclusion and exclusion criteria, 2 researchers will read the title and abstract of the literature to exclude the literature that obviously does not meet the inclusion criteria, and then obtain and read the full text of the literature, further exclude the literature that does not meet the inclusion criteria or have reasons for exclusion.

Two researchers will extract the data of the included studies, including the basic information of patients, the baseline of patients’ diseases, intervention/control measures and their treatment courses, outcome indicators, etc.

In case of disagreement, they will consult with a third researcher.

### 2.5 Risk of bias assessment

According to the Cochrane Handbook,^[18]^ the 2 researchers independently evaluated the quality of the input articles from 7 aspects. For each item, the correct use of method is low risk, unclear method is unclear risk, and incorrect or unused method is high risk. Two researchers will completed and cross checked independently, If there is a disagreement, they will discuss with a third researcher.

### 2.6 Statistical analysis

Markov chain Monte Carlo (MCMC) is a sampling method, which is used to solve the random sampling simulation problem of distribution that is difficult to directly sample. STATA14.0 software and MCMC method will be used for Bayesian meta-analysis to extract data from included studies. We will use 3 Markov chains to simulate, and set the number of iterations to be 50,000.

STATA14.0 will be used to draw the reticular diagram to show the comparison of different interventions more vividly. we will calculate RoR and its corresponding 95% confidence interval (CI). The closer the lower limit of 95%CI is to 1, the better the consistency. When the RoR is about 1, fixed effect model will be adopted. Otherwise, random effect model will be adopted. We will use odds ratio (OR) and 95%CI (*P* < .05) to express Dichotomous data, rank the efficacy of various interventions with WinBUGS1.4.3^[19]^ and record the area under the curve.

### 2.7 Assessment of heterogeneity

If *P* > .10 and *I*^2^ < 50%, Fixed-effect model will be used, otherwise, we will explore the sources of heterogeneity, and if we can’t find it, random effect model will be used. If the clinical heterogeneity is large and the results can not be combined, descriptive analysis will be carried out.

### 2.8 Subgroup analysis and sensitivity analysis

If there is enough data, we will consider grouping analysis. Sensitivity analysis can also be understood as robustness analysis, which is an important method to evaluate the robustness and reliability of the combined results of meta-analysis. The common sensitivity analysis method is exclude each study one by one and then merge the effects. After excluding 1 article, if the heterogeneity changes, we think that the study may be the source of it.

### 2.9 Evaluation of publication bias

We will establish a coordinate system with CIVIQ, VCSS and serum TGF-β1 levels as indicators, and draw a inverted funnel diagram in it. If the inverted funnel plot is symmetrical, it suggests that the possibility of publication bias is minimal.

### 2.10 Grading the quality of evidence

We will use the Grading of Recommendations Assessment, Development and Evaluation (GRADE) framework^[20,21]^ to appraise the quality of evidence derived from the network and pairwise meta-analysis.

## 3 Discussion

Lipodermatosclerosis, which is called skin arthralgia in TCM, is characterized by limited inflammation and fibrosis of the skin and subcutaneous tissue. Its large harmfulness and wide incidence rate make patients suffer from enormous economic and living burden. The etiology and pathogenesis of LDS is nothing more than the deficiency of Qi, blood, liver and kidney. TCM has a broad application prospect in the treatment of LDS by implementing different treatment principles and giving different prescriptions in different stages of disease. According to Professor Xuan, the basic pathogenesis of LDS is skin dystrophy caused by deficiency of both liver and kidney, deficiency of both qi and blood, cold coagulation and blood stasis, and obstruction of meridians. The essence of the disease is deficiency in origin and excess in superficiality. Deficiency in the origin is deficiency of qi, blood, liver and kidney, while excess in superficiality is cold coagulation and blood stasis. Professor Xuan^[22]^ proposed the treatment principle of nourishing liver and kidney, focusing on Supplementing Qi and blood, warming yang and dispersing cold, promoting blood circulation and dredging collaterals.

It is well known that TGF-β1 plays an vital role in the development of LDS. Therefore, down-regulation of TGF-β1 expression can reduce fibrosis, which plays an essential role in the treatment of LDS. Previous studies have shown that many herbs in the Chinese Pharmacopoeia, such as SophoraflavescensAit, Phellodendron chinense, Forsythiae fructus, Licorice, Cortex Moutan, Red Peony Root, and Mongolian Dandelion, has the clinical effect of lower TGF-β1. Studies demonstrate that Oxymatrine protects against myocardial fibrosis through the inhibition of TGF-β1.^[23]^ Phellodendron chinense extracts significantly decrease the level of TGF-β1 relative to reference group.^[24]^ Forsythiae fructus extracts have the effect of reducing TGF-β1 production in neutrophils and monocyte/macrophage cells.^[25]^ The aqueous extracts of licorice alleviate the expression of TGF-β1 and TNF-a in mice.^[26]^ Cortex Moutan extracts inhibit the level of TGF-β1 in a dose and time dependent manner, showing inhibition for pulmonary fibrosis.^[27]^ Red Peony root fumigation can reduce the level of TGF-β1, improve the state of inflammation in the treatment of chronic pelvic inflammatory disease.^[28]^ Diosgenin, a component of Mongolian dandelion, inhibits the proliferation of rat Mesangial cells dose-dependently, and down-regulates the expression of TGF-β1.^[29]^ Because TGF-β1 is the principal inducement of the pathogenesis of LDS, TCM can help to reduce the level of TGF-β1, so we can think that TCM has a broad prospect in the treatment of LDS.^[30]^

In addition, Chinese herbal pieces are processed into pill, Powder, Ointment and other different dosage forms by scientific processing technology, which is convenient to take, and has good curative effects. As a clinical irreplaceable treatment method for LDS, TCM has not been compared of the advantages and disadvantages so far. Therefore, we will use NMA to compare the efficacy of TCM for LDS systematically, so as to provide a reliable theoretical basis for the clinical application of TCM in the treatment of LDS.

## Author contributions

**Conceptualization:** Mengmeng Du, Xiaohua Shi.

**Data curation:** Mengmeng Du, Xiaohua Shi.

**Formal analysis:** Mengmeng Du, Ming Liu, Xiaohua Shi.

**Funding acquisition:** Yudong Zhang.

**Investigation:** Mengmeng Du.

**Methodology:** Mengmeng Du, Ming Liu, Xiaohua Shi.

**Project administration:** Mengmeng Du.

**Resources:** Mengmeng Du.

**Software:** Mengmeng Du, Ming Liu, Xiaohua Shi.

**Validation:** Mengmeng Du.

**Visualization:** Mengmeng Du.

**Writing – original draft:** Mengmeng Du, Xiaohua Shi.

**Writing – review & editing:** Mengmeng Du, Ming Liu.
